# Gold Foil

**Published:** 1870-01

**Authors:** W. H. Shadoan


### GOLD FOIL.

BY W. H. SHADOAN, D.D.S.

It is now about fifteen years since I filled the first tooth. For several years I was as a great many are now, content with simply producing a filling that would *stay in*, that being then all that was thought necessary. Then one kind of foil was just as acceptable as another. But as I began to properly appreciate the situation, I began to ask myself, "is this all that is necessary?" I began to see my fillings turn *black* around them, and on taking them out found that the decay was hardly checked in its progress. Now, while I am willing to acknowledge this, I am constantly being called on by patients to have work done, and it seems to me that three out of every five fillings that comes to me from other Dentists are in precisely this condition. Occasionally I find one of my fillings failing. There are various causes for this, but when I find teeth filled with gold, and it is so soft and spongy it may be penetrated with a common brass pin, I know that the Dentist is at fault.

But, I did not intend to write a treatise at this time on filling teeth. I only wish to call the attention of the profession to a gold that I find is little known as a general thing. I commenced the practice by using Morgan's gold foil, and Jones', White's, McCardy's, J. B. Dunlevy's and Chas. Abby & Sons. I soon found that of these I prefered Dunlevy's to any of the others. It was more uniform and less *crankey*, as one of my neighbors say when he finds gold that won't work smoothly.

I continued the use of Dunlevy's almost exclusively for about ten years. Since then, I have used a foil known as "Eaking's Foil," by W. H. Eaking, of Philadelphia, but soon found it about like Abby & Sons. I have used considerable of Leslie's gold foil, but found nothing very superior in it, although it worked very well in part, yet it was not free from some of the objections of other foils. I have used some foil made by Mr. Miller which was very fine, also some by M. M. Johnson & Co., N. Y. This foil works very well so far as I have had an opportunity of testing it.

There are other foils all of which have their favorites. Of the foil I have used until recently, I found Dunlevy's to excel all others. It was more uniform as to tenacity, softness, and adhesiveness. And if all foils worked with the same freedom as his, we would have little to complain of.

There is one make that I have not yet spoken of that claims especial notice. Sometime last winter, or early in the spring, Dr. W. G. Redman, of our city, gave me a specimen of gold foil, made by J. M. Ney & Co., of Hartford, Conn., which he said pleased him very much indeed. He uses soft foil altogether, and he said it works into the cavity more like wax than anything he could compare it to. He said the only fault he had with it was, it seemed he never could get a cavity full. It condensed so much. I remarked, that I liked it for that. If he could get more of Ney's gold into a cavity than any other it argued one essential thing, that was, that the particles were susceptible of a better arrangement than that of other foils. I took some of his foil and tried it, and found it as he said—soft and ductile.

Now, there is one marked difference in Dr. Redman's mode of operating and mine. He uses soft foil; I use the adhesive. Ney's is as soft as buckskin, and by annealing it becomes quite adhesive. These are grand points. A foil that is sufficiently soft for one man, and yet adhesive enough for my hand, are qualities worthy the consideration of all. I find that after filling a tooth with this foil, that it does not have that hard unyielding surface usually found in foils of other makers.

I have been using this foil for nearly a year, and in that time I have used several ounces, and have yet to find the first book or part of book that does not work well. In a word, I am perfectly delighted with it. When I get to working it, I sometimes wish the whole Dental profession could witness it work. Try it all of you who have not already done so. As to numbers, I am using No. 3, pretty generally. Have tried some of the heavier foils recently. Am very well pleased with them since witnessing their use by Dr. Atkinson last summer. They are good in large cavities.

